# Chagas disease and systemic autoimmune diseases among Bolivian patients in Switzerland

**DOI:** 10.1590/0074-02760170383

**Published:** 2018-02-05

**Authors:** Yves Jackson, Drenusha Vieira de Mello Pula, Axel Finckh, Carlo Chizzolini, François Chappuis

**Affiliations:** 1 Geneva University Hospitals and University of Geneva, Division of Primary Care Medicine, Geneva, Switzerland; 2 University of Geneva, Institute of Global Health, Geneva, Switzerland; 3 Geneva University Hospitals and University of Geneva, Division of Rheumatology, Geneva, Switzerland; 4 Geneva University Hospitals and University of Geneva, Division of Immunology, Geneva, Switzerland; 5 Geneva University Hospitals and University of Geneva, Division of Tropical and Humanitarian Medicine, Geneva, Switzerland

**Keywords:** Chagas disease, migrants, systemic autoimmune diseases, cardiomyopathy, autoimmunity

## Abstract

**BACKGROUND:**

Chronic cardiomyopathy occurs in 20-40% of the patients with Chagas disease. Autoimmune mechanisms may contribute to its pathogenesis. We diagnosed several cases of systemic autoimmune diseases among Bolivian migrants in Geneva with a high prevalence of Chagas disease.

**OBJECTIVES:**

We tested the hypothesis of a clinical association between systemic autoimmune diseases and Chagas disease, particularly with the development of cardiomyopathy.

**METHODS:**

We retrospectively searched the medical records of all Bolivian patients visiting Geneva University Hospitals between 2012 and 2015 for diagnosis of Chagas disease or systemic autoimmune diseases.

**FINDINGS:**

Of the 2,189 eligible patients, 28 [1.3%; 95% confidence interval (CI) = 0.9-1.9%] presented with systemic autoimmune disease. The Chagas status was known in 903 (41.3%) patient, of whom 244 (27.0%; 95% CI = 24.2-30.0%) were positive. Eight (28.6%; 95% CI = 15.3-47.1%) of the 28 cases of systemic autoimmune disease had Chagas disease. We found no association between both entities (p = 1.000) or with Chagasic cardiomyopathy (p = 0.729). Moreover, there was no evidence of a temporal relationship between antiparasitic chemotherapy and the development of systemic autoimmune diseases.

**CONCLUSIONS:**

Our results do not support a clinical association between chronic Chagas disease and systemic autoimmune diseases. However, prospective studies in areas endemic for Chagas disease should better assess the prevalence of systemic autoimmune diseases and thus a possible relationship with this infection.

Infection with *Trypanosoma cruzi*, the protozoan agent of Chagas disease, affects 6-8 million people, mostly in the Americas. International mobility has driven its emergence in North America and Europe, with Europe may host 68,000 to 122,000 cases (Basile et al. 2011). Unless treatment is promptly initiated after the initial exposure, the infection becomes chronic and leads to organ damage over decades, including potentially fatal cardiomyopathy, in 20-40% of patients (Bern 2015). The complex and interacting mechanisms leading to cardiomyopathy are not well understood. The current hypothesis includes parasite-dependant inflammation and a Th1-Th2 immune-reaction imbalance acting as prominent factors (Rassi Jr et al. 2017). Microvascular and neurogenic disturbance probably play a minor role. Some evidence points to a possible role of autoimmune mechanisms, with ongoing debate about the extent of such factors along with parasite-driven immunopathology (Bonney & Engman 2015). Indeed, various *T. cruzi* antigens cross-react with human components, notably cardiac proteins (Marin-Neto et al. 2007). Experimental studies have demonstrated that the induction of polyantigenic autoimmune activation generated by molecular mimicry in association with an enhanced Th1 response could lead to myocardial damage in animals and humans (Girones et al. 2001, Cunha-Neto et al. 2006). However, compelling evidence of the clinical significance of such mechanism in human Chagas cardiomyopathy is lacking (Bonney & Engman 2015).

Although the immunomodulatory mechanisms in chronic helminth infections are associated with a reduced risk and severity of allergic, chronic inflammatory, and systemic autoimmune diseases, no such mechanism has been found in protozoan infections (Tracey et al. 2016, Sarter et al. 2017).

Since the mid-2000's, an increasing number of Chagas disease cases have been identified among Latin-American immigrants in Switzerland (Jackson & Chappuis 2011). A study in Geneva showed a prevalence of 12.8% among Latino immigrants and 26.2% among Bolivians (Jackson et al. 2010a). In Europe, Bolivians account for more than 90% of the caseload (Requena-Méndez et al. 2015). During the same period, an unexpectedly high number of cases of systemic autoimmune disease were diagnosed among Bolivian patients consulting at the Geneva University Hospitals (HUG). Notably, a case of antisynthetase syndrome was diagnosed in a 43-year-old woman with chronic Chagas disease whose initial symptoms started five months after terminating nifurtimox antiparasitic therapy. We aimed to test the hypothesis of a statistical and clinical association between chronic *T. cruzi* infections, notably complicated by cardiomyopathy, and systemic autoimmune diseases in a population with a high prevalence of Chagas disease.

## MATERIALS AND METHODS

*Setting* - In 2012, 950 Bolivians were registered as residents in Geneva Canton. However, considering the large proportion of Bolivians living without residency permits in Switzerland, their total population was estimated at 3,000 (Morlok et al. 2015). The Geneva University Hospitals (Hôpitaux Universitaires de Genève, HUG) is the only public healthcare facility in Geneva Canton and acts as the main port of entry into medical care for recently arrived immigrants. It is the reference centre for the management of Chagas disease and systemic autoimmune diseases in the population. Since 2008, all pregnant Latin American women are screened for Chagas disease during their prenatal visits at HUG. Screening is proposed to all other patients in presence of clinically suggestive signs or symptoms or epidemiological risk factors. There is no systematic screening for systemic autoimmune diseases and investigations leading to a definitive diagnosis are initiated based on clinical suspicion.

*Design and patients* - This retrospective study included all adult (18 years and older) patients originating from Bolivia and attending the Geneva University Hospitals between January 1, 2012, and December 31, 2015.

*Source of data* - We searched the HUG administrative databases to identify eligible patients based on their nationality and age at the time of their first contact with the HUG. Electronic medical records and clinical databases of rheumatology, dermatology, immunology, and tropical diseases units were searched to identify Chagas disease and systemic autoimmune diseases cases.

*Definition of systemic autoimmune disease cases* - The following conditions were considered indicative of systemic autoimmune disease and were included in the analysis: systemic lupus erythematosus, Sjögren syndrome, systemic sclerosis, polymyositis, dermatomyositis, antisynthetase syndrome, rheumatoid arthritis, adult-onset Still's disease, undifferentiated systemic autoimmune disorder, and overlap syndrome. All cases met the most current diagnostic and classification criteria and were confirmed by a senior consultant in immunology, dermatology, or rheumatology.

*Definition of Chagas disease cases and staging* - The diagnosis of Chagas disease was considered in the presence of at least two serological tests positive for *T. cruzi* infection. In accordance with World Health Organization (WHO) recommendations, we use different tests based on different antigens or techniques. Our diagnostic algorithm involves the use of the Chagas Stat-Pak (Chembio Diagnostic Systems), a rapid immunochromatographic test, and two enzyme-linked immunosorbent assays (ELISAs) (Bio-Mérieux Elisa cruzi and Biokit Bioelisa Chagas). Staging of infection was assessed by reviewing the clinical, electrocardiographic (ECG), and imaging results. In case of abnormalities in the ECG or clinical symptoms suggestive of Chagas disease cardiomyopathy, 24-hour Holter test and cardiac ultrasonography were performed. Patients presenting with persistent upper or lower digestive tract symptoms suggestive of Chagasic digestive organ damages underwent barium studies. The absence of clinical symptoms and normal ECG findings prompted the diagnosis of the indeterminate form of the chronic phase.

*Statistics* - Categorical variables are presented as absolute numbers and proportions with 95% confidence intervals (95% CIs) and continuous variables are presented as means and standard deviations (SDs). Proportions were compared using chi-square Fisher exact tests for categorical variables and Student's-t tests for continuous data. The significance level was set at 0.05. The association between Chagas disease (or Chagasic cardiopathy) and systemic autoimmune diseases was assessed by comparing the respective prevalence among individuals with or without systemic autoimmune disease.

*Ethics* - This study received ethical clearance from the Geneva Canton Research Board (project 07-285).

## RESULTS

A total of 2,189 Bolivian patients were registered in the HUG databases during the study period, of whom 1,662 (75.9%) were female. The mean age at first contact with HUG was 37.1 (SD: 11.1) years.

*Chagas disease* - The serological status was known for 903 (41.3%) patients. Of these, 244 (27.0%; 95% CI: 24.2%-30.0%) were positive, of whom 212 (84.8%) were female. All were in the chronic phase of infection, 199 (81.6%) with the indeterminate form, 43 (17.6%) with cardiomyopathy, and two (0.8%) with digestive tract involvement. According to epidemiological and clinical data, we estimated that all patients had likely been infected during infancy or childhood. One hundred forty-two (58.2%) of the Chagas-positive patients had received antiparasitic chemotherapy, all of them at HUG. Chagas disease was significantly associated with older age (p < 0.001) but not with gender (p = 0.449).

*Systemic autoimmune diseases* - Overall, 28 (1.3%; 95% CI: 0.9%-1.9%) of the 2,189 patients presented with a systemic autoimmune disease (Table). The most frequent diagnoses were rheumatoid arthritis (n = 10), systemic lupus erythematosus (n = 4), undifferentiated systemic autoimmune disorder, Sjögren syndrome and adult-onset Still's disease (n = 3 each). All patients except for one (96.4%) were female with a mean age of 41.1 (SD: 10.5) years at the first onset of the systemic autoimmune disease symptoms.

**TABLE:** Description and Chagas disease status of 28 Bolivian patients with systemic autoimmune diseases in Geneva, Switzerland

Autoimmune systemic diseases				Chagas disease			
Gender	Type	Year of onset	Age at onset	Serological status	Cardiomyopathy	Antiparisitic chemotherapy	Year
F	OS (LES/SSc)	2008	34	positive	no	no	-t
F	RA	2004	48	positive	no	no	-
F	DM	2008	45	positive	yes	yes (BZ)	2005
F	SLE	2013	65	positive	no	yes (BZ)	2013
F	ASS	2008	54	positive	no	yes (BZ)	2009
F	SS	2006	42	positive	yes	yes (BZ)	2013
F	AOSD	2013	31	positive	no	yes (BZ + NIF)	2011
F	ASS	2009	44	positive	no	Yes (NIF)	2008
F	RA	1997	40	negative			
F	RA	2015	60	negative			
F	OS (RA/SS)	2013	37	negative			
F	RA	2005	38	negative			
F	RA	1995	31	negative			
F	SS	2014	54	negative			
M	RA	1988	28	negative			
F	SLE	2009	44	negative			
F	RA	2015	34	negative			
F	SD	2013	28	negative			
F	RA	2013	31	negative			
F	USAD	2013	37	negative			
F	SLE	2010	21	negative			
F	SLE	2007	33	negative			
F	SS	2011	39	negative			
F	RA	2009	48	negative			
F	USAD	2011	50	negative			
F	RA	2008	36	negative			
F	USAD	2006	42	negative			
F	AOSD	2014	56	negative			

ASS: antisynthetase syndrome; DM: dermatomyositis; OS: overlap syndrome; RA: rheumatoid arthritis; AOSD: adult-onset still disease; SLE: systemic lupus erythematosus; SS: Sjögren syndrome; USAD: undifferentiated systemic autoimmune disorder; BZ: benznidazole; NIF: nifurtimox.

*Systemic autoimmune diseases and Chagas disease* - Eight (28.6%; 95% CI: 15.3-47.1%) of the 28 patients with systemic autoimmune disease were diagnosed with Chagas disease, all of them female. The systemic autoimmune diseases among Chagas disease cases included antisynthetase syndrome (n = 2), overlap syndrome, dermatomyositis, systemic lupus erythematosus, Sjögren syndrome, rheumatoid arthritis, and adult-onset Still's disease (n = 1 each). At the initial onset of the systemic autoimmune disease symptoms, six patients presented with the indeterminate form of the chronic phase of Chagas disease and two patients presented with cardiomyopathy (one with cardiomegaly and mild left ventricle failure, one with ST-T changes on ECG and normal ventricular function).

The two cases of antisynthetase syndrome occurred among patients with Chagas disease, one before and one after receiving antiparasitic chemotherapy. Only one in the 10 rheumatoid arthritis cases and none of the three cases of undifferentiated systemic autoimmune disorder occurred among Chagas disease patients.

*Statistical and clinical association* - The presence of systemic autoimmune diseases was not statistically associated with Chagas cardiomyopathy (p = 0.729) and we found no overall association between Chagas disease and systemic autoimmune diseases (p = 1.000). Among the 28 cases of systemic autoimmune diseases, Chagas disease patients were older than non-Chagas disease ones (mean 45.4 versus 39.4 years) but this difference was not significant (p = 0.175).

In total, six patients received antiparasitic chemotherapy, including one who had experienced *T. cruzi* reactivation after initiation of immunosuppressive therapy. In three of these six cases, antiparasitic treatment preceded the onset of the symptoms of systemic autoimmune disease. One patient had received benznidazole (60 days), one nifurtimox (60 days), and one benznidazole (10 days) followed by nifurtimox (20 days) with poor tolerance resulting in premature discontinuation. The time interval between treatment initiation and the onset of the first systemic autoimmune disease symptoms ranged from 5 to 36 months. The systemic autoimmune diseases that occurred after antiparasitic chemotherapy included antisynthetase syndrome, dermatomyositis. and adultonset Still's disease (n = 1 each).

## DISCUSSION

This is the first study to explore the prevalence of systemic autoimmune diseases and the association between chronic Chagas disease and systemic autoimmune diseases in Bolivian migrants in Europe. We found that 27% of the participants had Chagas disease and 1.3% had a systemic autoimmune disease. The prevalence of Chagas disease and the proportion of disease at an advanced phase as attested by the presence of cardiomyopathy are consistent with findings in migrants from this age group in other European countries (Basile et al. 2011, Requena-Méndez et al. 2015). This finding confirms that preventive and clinical management strategies should prioritize the targeting of this group.

We described 28 cases of systemic autoimmune diseases, including 10 (0.4% of the total cohort) with rheumatoid arthritis. There are no data about rheumatoid arthritis and other systemic autoimmune diseases prevalence in Bolivia to compare with our results. In Argentina, the rheumatoid arthritis prevalence in the general population ranges from 0.2 to 0.9%, and up to 2.4% among indigenous groups (Quintana et al. 2016). The limited epidemiological evidence about systemic autoimmune diseases in Latin American shows that patients with systemic lupus erythematosus have worse clinical outcome when originating from poor socioeconomic backgrounds, which is similar to Chagas disease (Briceño-León & Galván 2007, Pons-Estel et al. 2015). The development of regional registries for systemic autoimmune diseases is likely to provide better understanding of the real burden of rheumatoid arthritis and other autoimmune diseases in this population and may contribute to explorations of the epidemiological association with locally endemic chronic infectious diseases such as Chagas disease (Pons-Estel et al. 2012). Patients with systemic autoimmune diseases are likely to receive immunosuppressive therapies at some stage and thus may reactivate clinically silent chronic parasitic infections such as strongyloidiasis and Chagas disease, resulting in high morbidity and mortality. It is, therefore, essential to systematically screen for such infections in all patients with systemic autoimmune diseases.

We found no evidence of a statistical and clinical association between systemic autoimmune diseases and (i) Chagas disease, (ii) Chagasic cardiomyopathy, or (iii) antiparasitic treatment. Autoimmune diseases have been previously reported in patients with *T. cruzi* or other protozoan infections but never within a causal perspective or in relation to antiparasitic treatment (Xynos et al. 2009, Pinazo et al. 2013). Previous case reports focused on the risks and clinical management of *T. cruzi* reactivation following the initiation of immunosuppressive therapies (Pinazo et al. 2013).

The relationship between antiparasitic treatment of Chagas disease and immune activation is not well understood. We previously described three drug rash with eosinophilia and systemic symptoms (DRESS) cases among 81 patients treated with nifurtimox (onset after treatment initiation: 12 to 63 days) (Jackson et al. 2010b). To the best of our knowledge, none of those cases subsequently developed a systemic autoimmune disease. A recent case series reported six episodes of oligo- or polyarthritis associated with benznidazole treatment (onset: 25 to 42 days) (Aldasoro et al. 2015). One case presented serological and clinical evidence evocative of rheumatoid arthritis which could not be formally proven. All cases of arthritis resumed within 7-62 days following a short course of nonsteroidal anti-inflammatory drug (NSAID) therapy and without relapse. Our results do not support the hypothesis that antiparasitic chemotherapy may act as a trigger for the development of systemic autoimmune diseases; however, larger studies systematically recording tolerance to antiparasitic treatment with extended follow-up may be useful to better assess this possible association.

Eight (28.6%) of the 28 cases of systemic autoimmune diseases were infected with *T. cruzi*. Only 15 cases of Chagas disease and systemic autoimmune diseases have been reported so far (Cossermelli e al. 1978, Barousse et al. 1980, dos Santos-Neto et al. 2003, Pinazo et al. 2010, 2013, Burgos et al. 2012, González-Fontal et al. 2013). All except for two were systemic lupus erythematosus. We provide here the first report of the coexistence of Chagas disease with a range of other autoimmune diseases, notably antisynthetase syndrome and rheumatoid arthritis. While there have been suggestions that auto-antibodies reacting against similar epitopes in systemic lupus erythematosus and Chagas disease could be associated with organ damage, recent studies showed that antibodies directed against the ribosomal P protein in humans and in *T. cruzi* differ in structure and were reactive to distinct peptides (Grippo et al. 2011, Abraham & Derk 2015). Some evidence points to a causal link between chronic infections and the development of rheumatoid arthritis. Indeed, chronic infection with *Porphyromonas gingivalis*, a leading pathogen in periodontitis, leads to autoimmune response to the host's citrullinated epitopes and the risk of developing clinical rheumatoid arthritis (Koziel et al. 2014). More research is needed to assess a similar link with *T. cruzi*.

Our study has several limitations: (a) a selection bias due to the hospital-based recruitment that may not reflect the composition and burden of disease of the general population. However, the high proportion of participants with regard to the estimated local population suggests that this cohort could fairly represent Bolivian immigrant communities in Switzerland and, by extension in Europe; (b) measurement bias pertaining to the absence of the systematic screening of systemic autoimmune diseases and Chagas disease in all eligible patients due to the retrospective study design as well as the risk of missed diagnoses in paucisymptomatic patients with systemic autoimmune diseases; (c) the low number of cases, which limited the statistical power to calculate the measures of associations.

In conclusion, we described a series of systemic autoimmune diseases cases occurring among patients chronically infected with Chagas disease. The absence of statistical evidence of an association does not mean the absence of such an association considering the limited sample size in the present study. Our findings support the need for prospective studies in areas endemic for Chagas disease to better assess the prevalence of systemic autoimmune diseases and its possible co-occurrence with *T. cruzi* infection.
